# Sotagliflozin versus dapagliflozin to improve outcome of patients with diabetes and worsening heart failure: a cost per outcome analysis

**DOI:** 10.3389/fphar.2024.1373314

**Published:** 2024-04-17

**Authors:** Weichen Zhang, Meichen Yu, Guohua Cheng

**Affiliations:** Department of Pharmacy, Jinan University, Guangzhou, China

**Keywords:** sotagliflozin, dapagliflozin, heart failure, outcome analysis, diabetes

## Abstract

**Background and aim:**

Dapagliflozin inhibits the sodium-glucose cotransporter protein 2 (SGLT-2), while sotagliflozin, belonging to a new class of dual-acting SGLT-1/SGLT-2 inhibitors, has garnered considerable attention due to its efficacy and safety. Both Dapagliflozin and sotagliflozin play a significant role in treating worsening heart failure in diabetes/nondiabetes patients with heart failure. Therefore, this article was to analyze and compare the cost per outcome of both drugs in preventing one event in patients diagnosed with diabetes-related heart failure.

**Method:**

The Cost Needed to Treat (CNT) was employed to calculate the cost of preventing one event, and the Number Needed to Treat (NNT) represents the anticipated number of patients requiring the intervention treatment to prevent a single adverse event, or the anticipated number of patients needing multiple treatments to achieve a beneficial outcome. The efficacy and safety data were obtained from the results of two published clinical trials, DAPA-HF and SOLOIST-WHF. Due to the temporal difference in the drugs’ releases, we temporarily analyzed the price of dapagliflozin to calculate the price of sotagliflozin within the same timeframe. The secondary analyses aimed to assess the stability of the CNT study and minimize differences between the results of the RCT control and trial groups, employing one-way sensitivity analyses.

**Result:**

The final results revealed an annualized Number Needed to Treat (aNNT) of 4 (95% CI 3-7) for preventing one event with sotagliflozin, as opposed to 23 (95% CI 16-55) for dapagliflozin. We calculated dapagliflozin’s cost per prevented event (CNT) to be $109,043 (95% CI $75,856-$260,755). The price of sotagliflozin was set below $27,260, providing a favorable advantage. Sensitivity analysis suggests that sotagliflozin may hold a cost advantage.

**Conclusion:**

In this study, sotagliflozin was observed to exhibit a price advantage over dapagliflozin in preventing one events, cardiovascular mortality, or all-cause mortality in patients with diabetes.

## 1 Introduction

Heart failure (HF) is a complex clinical syndrome characterized by symptoms and/or signs resulting from structural and/or functional abnormalities of the heart. In most cases, it refers to a condition where the myocardial contractile function is diminished, leading to an inability to achieve the metabolic needs of the body. (Owan et al., 2006; Fonarow et al., 2007; Pitt et al., 2014). According to data from the American Heart Association (AHA) between 2017 and 2020, the total number of individuals aged 20 and above with heart failure was 6.7 million. It is projected that from 2012 to 2030, the incidence of heart failure (HF) will grow by 46%, with the overall proportion of heart failure patients rising from 2.4% to 3.0% over the course of a decade. This is expected to affect over 8 million adult patients. (Tsao et al., 2023). Diabetes stands as a significant risk element in the terms of heart failure, with approximately 30% of patients diagnosed with heart failure also having type 2 diabetes (T2DM). (Thrainsdottir et al., 2005; Lehrke and Marx, 2017). The data in a study from the National Hospital Quality Monitoring System (HQMS) revealed a rapid increase in the proportion of patients experiencing heart failure syndrome among those with both type 1 diabetes (T1DM) and T2DM in tertiary hospitals from 2013 to 2017. (Li et al., 2022).

Dapagliflozin falls within the category of medications known as sodium-dependent glucose transporters 2 (SGLT2) inhibitors. By the functions of SGLT2, the reabsorption of glucose was reduced in the renal tubules, resulting in a significant excretion of glucose in the urine and consequently reducing the levels of blood glucose. Additionally, the DAPA-HF study has established its efficacy in patients diagnosed with heart failure and reduced ejection fraction (McMurray et al., 2019) Patients treated with dapagliflozin had lowered the threats of worsening HF or/and cardiovascular-related death compared to those staying in the placebo group. However, concerns still exist regarding its cardiovascular (CV) safety. In type 2 diabetes patients with or at risk of atherosclerotic CV disease, dapagliflozin lowered the rates of CV death or hospitalization due to heart failure. (Cohen et al., 2023). Nevertheless, it did not significantly lower the incidence of major adverse cardiovascular events (MACE) compared to the placebo arm. (Wiviott et al., 2019; Palmer et al., 2021).

Sotagliflozin can block intestinal SGLT1 and renal SGLT2 glucose transporters, thereby reducing the absorption of glucose in the intestines and consequently reducing postprandial glucose and insulin concentrations, (Powell et al., 2020), By increasing the renal excretion of glucose, sotagliflozin lowers the level of glucose. Serving as an adjunct to insulin, the double-acting inhibitor of SGLT1 and SGLT2, sotagliflozin can enhance the manage of blood glucose levels in patients diagnosed with type 1 diabetes. Simultaneously, it reduces insulin dosage, promotes weight loss, significantly decreases the occurrence of severe hypoglycemia, and does not increase the probability of hypoglycemia occurrence. This enables more individuals with type 1 diabetes to meet therapeutic objectives without gaining weight within a specified period. (Sands et al., 2015; Buse et al., 2018; Danne et al., 2018; Danne et al., 2019; Rodbard et al., 2020). Additionally, oxidative stress, characterized by an excess of oxidative species, has been identified as one of the primary mechanisms contributing to the pathology of type 2 diabetes. (Andreadi et al., 2022).This process results in the production of advanced glycosylated end products (AGEs) or activation of the polyol pathway, which bind to receptors and induce the expression of adhesion molecules, impairing endothelial function and elevating the risk of cardiovascular disease. (Nakamura et al., 1993; Schmidt et al., 1995; Andreadi et al., 2023). The overproduction of reactive oxidative species (ROS) and reactive nitrogen species (RNS), or an imbalance between ROS and cellular antioxidants, contributes to the development of various diseases. (D'Autréaux and Toledano, 2007). Hyperglycemia free fatty acids (FFA), and pancreatic beta cell insulin release directly or indirectly induce the overproduction of ROS, disrupting intracellular homeostasis. (Andreadi et al., 2023). Animal model studies have identified SGLT-2i as a potent antioxidant drug capable of reducing oxidative sress by modulating the production of pro-oxidant enzymes such as Nox, eNOS, and xanthine oxidase. (Kawanami et al., 2017). Additionally, the study found that sotagliflozin significantly reduced cardiovascular outcomes compared to the control group, with a reduction from 76.3% to 51.0% in the primary outcome. (Docherty and McMurray, 2021; Andreadi et al., 2023). Among type 1 diabetes patients receiving insulin treatment, a higher percentage of patients in the sotagliflozin group achieved glycated hemoglobin levels below 7.0%, with no occurrence of severe hypoglycemia or diabetic ketoacidosis, compared to the placebo group. (Garg et al., 2017). The 2021 European Society of Cardiology (ESC) Diabetes Guidelines designate SGLT2 inhibitors as the primary recommended medication for individuals with diabetes who also have a concomitant high or very high cardiovascular risk, with a recommendation grade of ⅠA. (McDonagh et al., 2021). Recent research indicates that, compared to a placebo, the use of sotagliflozin has demonstrated significant efficacy in reducing the overall occurrence of cardiovascular-related deaths, hospitalizations due to heart failure, and emergency visits in individuals with diabetes and those with recently worsened heart failure. (Bhatt et al., 2021b).

The latest study indicates that the T_max_ of Sotagliflozin is 3 h, and the plasma protein binding rate is as high as 97.7%. In patients with Type 2 Diabetes Mellitus (T2DM) and normal renal function, sotagliflozin’s onset of action is rapidly absorbed, with T_1/2_ ranging between 13.5 and 20.7 h. This extended half-life can significantly enhance the duration of efficacy compared to the 13-h duration of dapagliflozin. Therefore, administering the drug directly before breakfast and once daily can maximize its effect. (Scheen, 2015; Garcia-Ropero et al., 2018).

Therefore, the aim of this study is to offer a prospective endpoint economically, comparing the cost of preventing heart failure in diabetic patients using sotagliflozin *versus* dapagliflozin for each outcome.

## 2 Methods

### 2.1 Data source

The original data for sotagliflozin were derived from the SOLOIST-WHF clinical trial, which was sponsored by Sanofi and Lexicon Pharmaceuticals. (Bhatt et al., 2021b). The dapagliflozin’s outcome data were rooted in the intervention group of adults with diabetes mellitus in the DAPA-HF study. (Petrie et al., 2020).

### 2.2 Primary outcome

The primary endpoint was the Cost Needed to Treat (CNT), preventing one event of hHF (Heart Failure hospitalizations) or the death of cardiovascular (composite outcome). (Mayne et al., 2006). This study was analyzed from the perspective of payment by the US healthcare payer.

### 2.3 Cost needed to treat/number needed to treat analysis

The Cost Needed to Treat (CNT) and the Number Needed to Treat (NNT) were introduced as an alternative way to demonstrate clinical benefit. (Thabane, 2003). The CNT was determined by the product of the annualized number needed to treat (aNNT) and the annual cost of treatment. (Mendes et al., 2017). Number Needed to Treat (NNT) signified the number of patients within a specific timeframe that one would need to treat to complete one extra study endpoint. The NNT was calculated as the reciprocal of the absolute risk reduction (ARR), presented as a decimal. We utilized drug costs in our analysis based on 75% of the US National Average Drug Acquisition Cost, as extracted in November 2023. (Data Medicaid, 2023).

### 2.4 Annualized number needed to treat analysis

The aARR represented the absolute difference between the annualized Absolute Risk (aAR) in the control group and the intervention group.

### 2.5 Secondary outcomes

Secondary outcomes included CNT to prevent one event of cardiovascular mortality (CV mortality) and all-cause mortality, considered as distinct clinical endpoints.

### 2.6 Sensitivity analysis

In order to assess the stability of the CNT study and reduce variations in outcomes between the RCT control and intervention groups, this study employed univariate sensitivity analysis. Analysis parameters included the event risk in the control arm of the RCTs and the annual cost associated with the interventions under compared.

To minimize the impact of drug variations in RCTs, this study simulates the annual event rates for each clinical trial drug in every clinical trial.

## 3 Results

### 3.1 Patient population

The patient demographics and heart failure with DM treatment modalities were effectively matched between the trial groups at the outset (McMurray et al., 2019; Bhatt et al., 2021b). A total of 2,747 subjects were included in this two randomized trials, as shown in Table 1. The medium follow-up was slightly shorter for Sotagliflozin (0.77 years) compared to Dapagliflozin (1.51 years). The medium age was 69 years in the Sotagliflozin group compared to 66.3 years in the Dapagliflozin group, indicating a minimal difference in mean age between the two groups of subjects. The majority of patients in both trials were white. The SOLOIST-WHF trial included patients with Hemoglobin of 7.1 and NT-proBNP (IQR) of 1816.8 pg/mL compared to Hemoglobin of 7.4 and NT-proBNP (IQR) of 1,479 pg/mL for DAPA-HF. Meanwhile, the median eGFR was 49.2% and the systolic blood pressure was 122 mmHg in the SOLOIST-WHF trial, compared to 63.9% and 121.4 mmHg in the DAPA-HF trial. Finally, the BMIs of the two groups of subjects equalized approximately.

**TABLE 1:** Key characteristics in the trial population.

Intervention trial	Sotagliflozin	Dapagliflozin
Number of patients with T2DM(%)	608 (100%)	2,139 (100%)
White (%)	93.3%	69.2%
Median follow-up (years)	0.77	1.51
Age (medium)	69	66.3
Female sex (%)	32.6%	22.3%
Medium Hemoglobin	7.1	7.4
Medium NT-proBNP(IQR)-pg/ml	1816.8	1,479
Medium eGFR (%)	49.2	63.9
Systolic BP	122	121.4
BMI	30.4	29.3

### 3.2 Annualized number needed to treat and cost needed to treat

**FIGURE 1:**
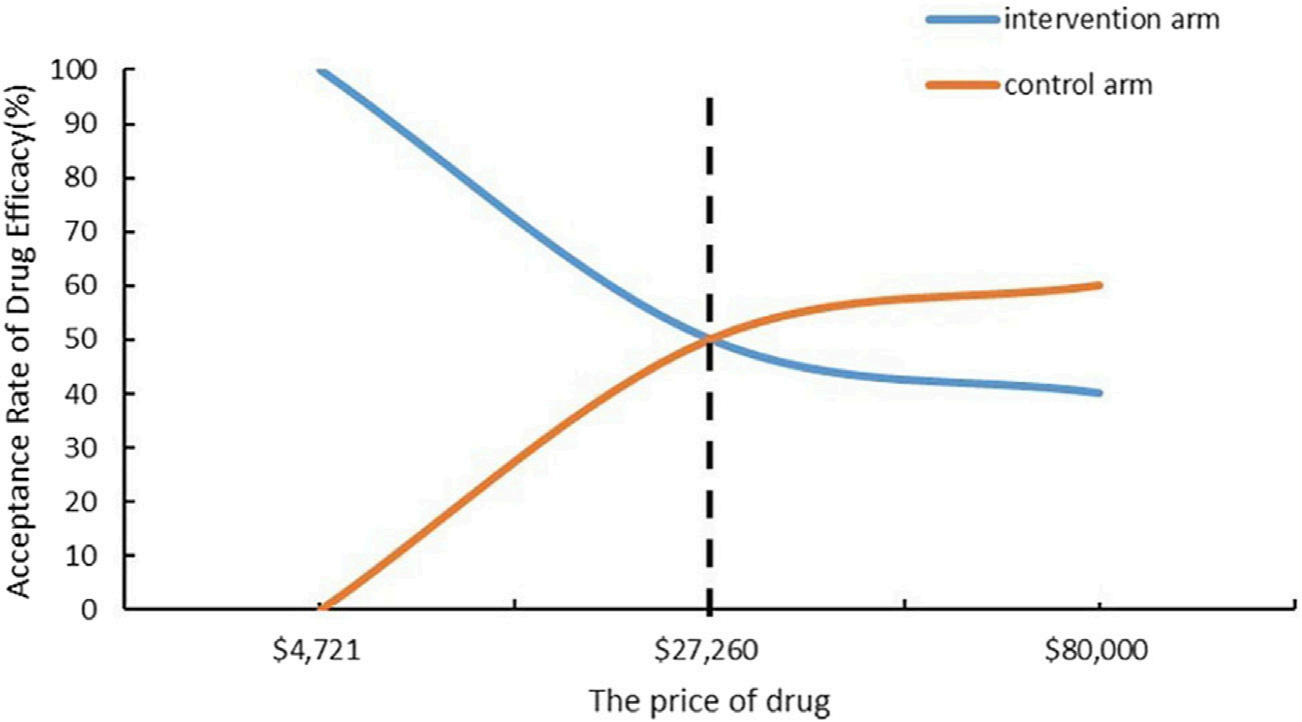
Cost-Acceptability Curve to prevent one event.

The computations of annualized NNT and CNT are shown in Table 2, listing the concrete calculation process. Figure 1 depicts the acceptable price curve for the simulation of sotagliflozin’s drug price, using 75% of the November 2023 updated NADAC for dapagliflozin as the baseline price.

**TABLE 2:** The calculations of the number and the cost needed to treat.

Parameter	Sotagliflozin	Dapagliflozin
Number of patients in the control arm	614	1,064
Patient years of therapy in the control arm	473	1,607
Number of events-control arm	355	271
Annualized event rate-control arm	75.05%	16.86%
Number of patients- intervention arm	608	1,075
Patient years of therapy- intervention arm	468	1,623
Number of events-intervention arm (95%CI)	238 (185–302)	203 (171–244)
Annualized event rate-intervention arm (95%CI)	50.85% (39.53–64.53%)	12.51% (10.54–15.03%)
Absolute event rate reduction (annualized) (95%CI)	24.2% (10.52–35.52%)	4.35% (1.83–6.32%)
Annualized number needed to treat (95%CI)	4 (3–7)	23 (16–55)
Annual drug cost	Figure 1	$4,741
Cost needed to treat to prevent one event (95%CI)	Figure 1	$109043 ($75856–260,755)

### 3.3 Secondary outcome analysis

**FIGURE 2:**
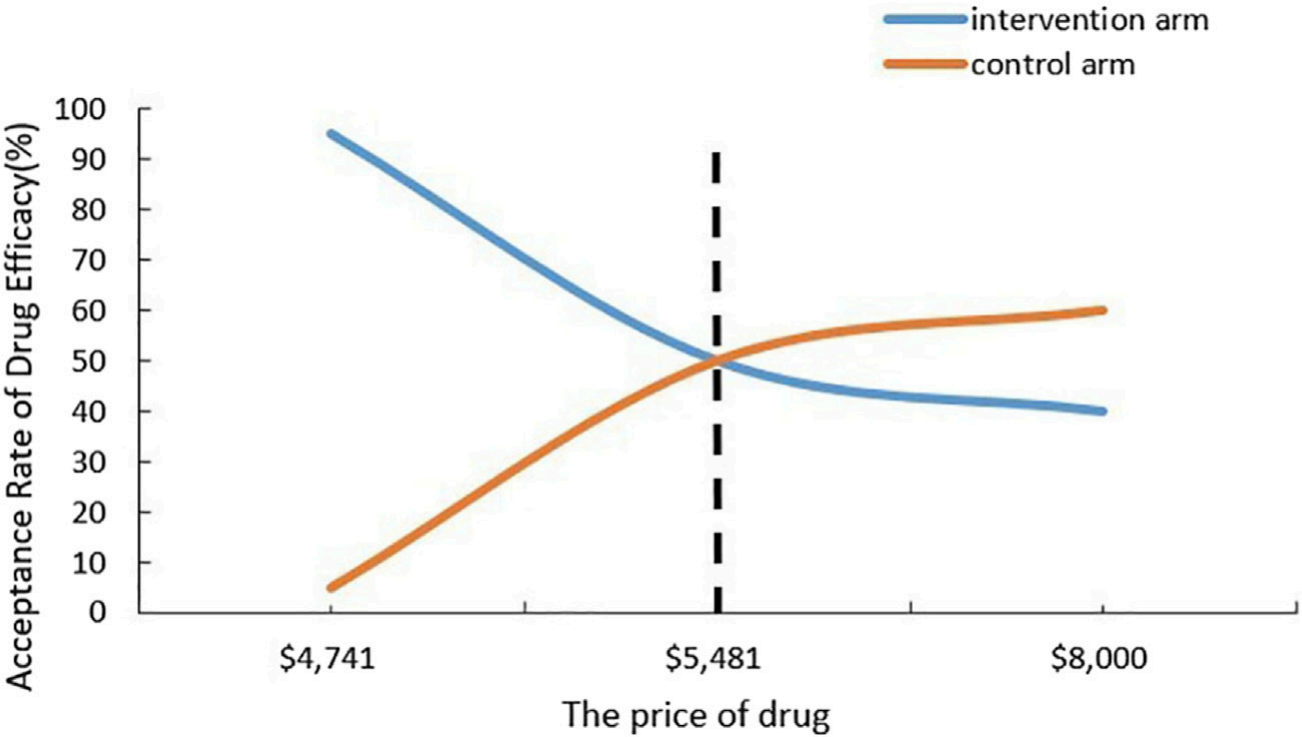
Cost-Acceptability Curve to All-cause mortality.

**FIGURE 3:**
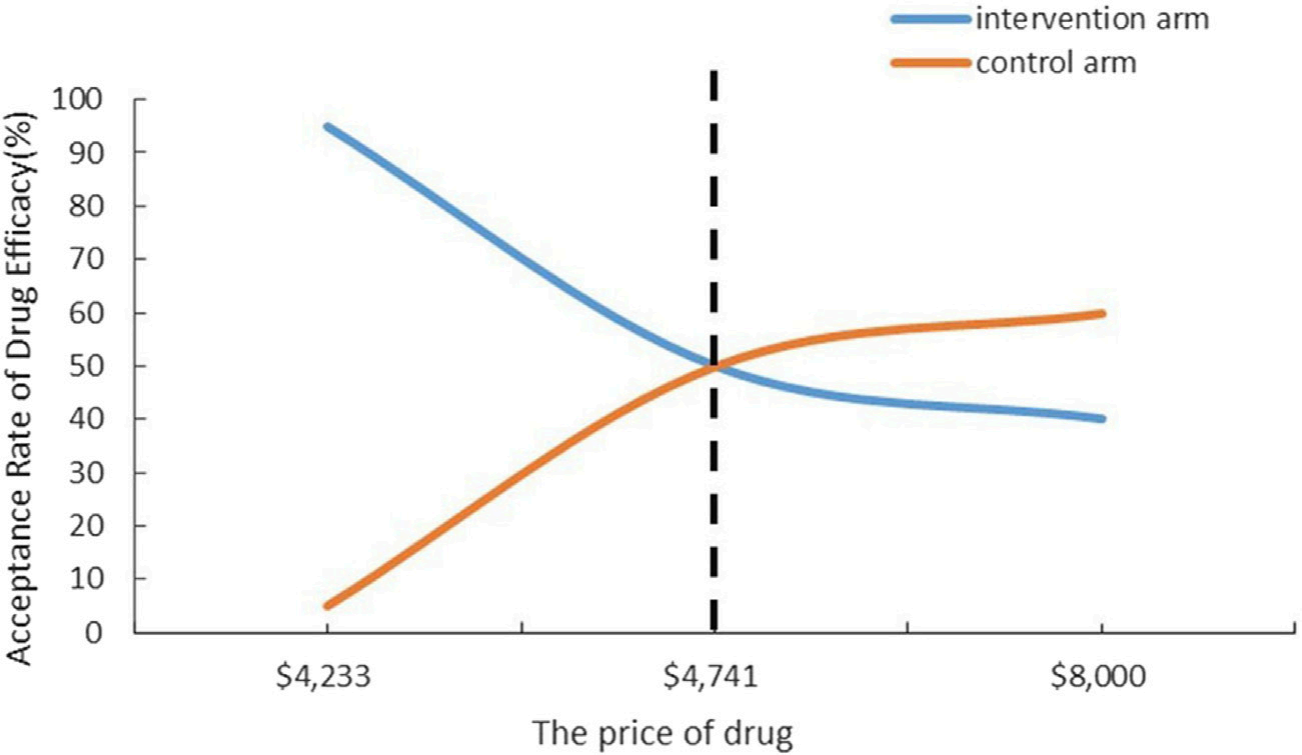
Cost-Acceptability Curve to CV mortality.

The CNT results of the secondary outcome are detailed in Table 3. Figures 2, 3 respectively present the results of simulating the NNT based on the calculated CNT for sotagliflozin and dapagliflozin, and the comparison between the two.

**TABLE 3:** Secondary of outcome analysis.

Outcome	Risk reduction		Annualized NNT		CNT	
	SOTA VS SOC	DAPA VS SOC	SOTA	DAPA	SOTA	DAPA
All-cause mortality (95%CI)	0.82 (0.59–1.14)	0.78 (0.63–0.97)	35 (16∼∞)	40 (24–238)	Figure 2	$189640 ($113784∼$1128358)
CV mortality (95%CI)	0.84 (0.58–1.22)	0.79 (0.63–1.01)	56 (19∼∞)	50 (29∼∞)	Figure 3	$237050 ($132748∼∞)

### 3.4 Sensitivity analysis

The outcomes of the sensitivity analysis, which involved simulating the use of different annualized event rates within the control arm according to the event rates in each of the trials, are presented in Table 4.

**TABLE 4:** Results of simulating the effect of intervention in the two RCTs.

	Value	CNT for sotagliflozin	CNT for dapagliflozin
Simulation of annualized event tare in the RCT control group	75.05% (as in SOLOIST-HF)	$18964 ($14223-$33187)	$85338 ($61633-$199122)
	16.86% (as in DAPA-HF)	$23705 ($18964-$56892)	Base-case:$109043 ($75856–260,755)

## 4 Discussion

The 2022 American College of Cardiology (ACC)/American Heart Association (AHA)/Heart Failure Society of America Guidelines for the Management of Heart Failure: (Failure, 2021): SGLT-2 inhibitors as the first choice for the therapy of heart failure, including dapagliflozin. However, as a new class of SGLT-1/SGLT-2 dual-acting inhibitors, the efficacy and safety of sotagliflozin have attracted much attention. Therefore, this study will give sound advice on clinical decision making from the following aspects.

This study determines that sotagliflozin is remarkably more effective in lowering the NNT compared to dapagliflozin for preventing one event. Refer to Table 2 for detailed information, the NNT for sotagliflozin was 4 (95% CI 3-7), whereas for the control group, it is 23 (95% CI 16-55). Notably, the NNT for the intervention group constitutes only 17.4% of that for the control group. In the calculation of the CNT, a sensitivity prediction analysis is employed, using the drug price as the baseline for the control drug and examining the indicators for the intervention group.

In Figure 1, the odds of cost acceptability for the intervention group approach 100% when both sotagliflozin and dapagliflozin are priced at $4,741. As the drug price rose to $27,260, patients’ acceptance of the prices for both drugs converged, with sotagliflozin being only 17.39% of the price of dapagliflozin. This implies that within the $4,741-$27,260 price range for sotagliflozin, choosing sotagliflozin is economically superior to selecting dapagliflozin.

This analysis suggests that the CNT is lower in the sotagliflozin group compared to the dapagliflozin group, indicating its superiority in terms of monetary value. In the secondary outcome analysis, we can compare sotagliflozin and dapagliflozin regarding all-cause mortality and cardiovascular mortality. For reducing all-cause mortality, the NNT for the sotagliflozin group is 35, slightly lower than the control group’s NNT of 40. Additionally, Figure 2 illustrates the economical price range for sotagliflozin, spanning from $4,741 to $5,481. This indicates that despite the price of sotagliflozin being higher than $4,741 but lower than $5,481, it is still considered economical. Nevertheless, for reducing cardiovascular mortality, the NNT of sotagliflozin is slightly higher than that of the dapagliflozin. The price sensitivity analysis reveals that sotagliflozin is considered economical only when priced below $4,741.

In the results of simulating the intervention effects in the two RCTs, the Cost-Effectiveness Ratio (CNT) for base treatment is $18,964 (95% CI $14,223-$33,187), compared to the dapagliflozin group’s CNT of $855,338 (95% CI $61,633-$199,122). In this analysis, the drug price of dapagliflozin is equated with that of sotagliflozin, demonstrating a significant reduction in drug purchase expenditures. Additionally, this analysis shows a substantial decrease in medication expenses when the drug price of dapagliflozin is equated with that of sotagliflozin. Similarly, with an annualized event rate of 16.86% in the control group, the CNT is $23,707 (95% CI $18,964-$5,689,892) in the sotagliflozin group compared to $109,043 (95% CI $75,856-$2,607,555) in the dapagliflozin group.

In summary, patients receiving sotagliflozin exhibited lower incidence rates of both primary and secondary outcome events compared to those in the control group. During the SOLOIST-HF trial, the overall incidence of CV death, urgent heart failure visits, and heart failure in the control arm was 76.4%. In the experimental arm receiving sotagliflozin, the overall incidence of events was 51.3%, representing a significant reduction of 25.1%. Breaking down the primary outcome measures, the event rate for cardiovascular death in the experimental group was 8.4% compared to 9.4% in the control group, indicating a 1% reduction in occurrence. The incidence of hospitalization due to heart failure events in the experimental arm (33.7%) was markedly lower than in the control arm (51.9%). Moreover, the occurrence rate for urgent heart failure visits was decreased by 5.2%.

Dual mechanism of action of sotagliflozin may have potential clinical advantages. The kidney plays a crucial role in the body’s glucose metabolism, and glucose transport in the body relies on sodium-dependent glucose transporter carriers (SGLTs). SGLT-2, primarily located in the S1 segment of the renal proximal tubule, functions as a low-affinity, high-capacity transporter. Meanwhile, its inhibitors protect pancreatic β-cell function. (Brunton, 2015). Consequently, it plays a significant role in glucose reabsorption. This phenomenon elucidates the ability of SGLT-2 inhibitors to effectively reduce blood glucose levels. Studies have found that genetic mutations in SGLT-1 can lead to severe diarrhea, even life-threatening. (van den Heuvel et al., 2002). It is probable that dual inhibitors targeting both SGLT1 and SGLT2 may provide vascular benefits similar to, or potentially surpassing, those of selective SGLT2 inhibitors. (Kashiwagi et al., 2015).Dapagliflozin is highly potent, reversible, and selectively inhibits sodium-glucose cotransporter-2, making it a widely used medication for treating type 2 diabetes mellitus. Moreover, dapagliflozin’s cost-effectiveness compared to similar medications may be substantial. (McEwan et al., 2020; Nguyen et al., 2023). Additionally, genital infections are more prevalent. (Dhillon, 2019). Significant barriers hinder the adoption of SGLT2 inhibitors. However, despite the benefits and guidelines provided by the Society of Cardiology, the rates of clinical prescribing are low. (Vardeny and Vaduganathan, 2019). This is primarily attributed to a lack of understanding of the medication, concerns about introducing confusion into diabetes care, and discomfort with prescribing diabetes medications. (Gao et al., 2020). According to a systematic review, sotagliflozin demonstrated significant reduction in cardiovascular mortality, hospitalizations, and urgent HF visits due to heart failure when compared to dapagliflozin. Conversely, dapagliflozin exhibited notably significant benefits in terms of cardiovascular mortality and the worsening heart failure. (Iyer et al., 2023).

Overall, the analysis of data indicates that the benefits of sotagliflozin on heart failure and blood glucose control across the entire spectrum of renal function can be summarized in two main aspects. Firstly, sotagliflozin significantly reduces the overall incidence of CV death, heart failure hospitalizations, and urgent heart failure visits; (Bhatt et al., 2021b);Secondly, as an oral double-acting inhibitor of SGLT-1/SGLT-2, sotagliflozin markedly lowers glycated hemoglobin (HbA1c) levels in patients with alleviate to moderately severe chronic kidney disease (CKD), demonstrating significant efficacy individual with CKD. (Bhatt et al., 2021a). Relevant studies have demonstrated that sotagliflozin prevents the onset of atrial arrhythmias by additional SGLT1 inhibition. (Bode et al., 2021). However, it is associated with an increase in diarrhoea, genital infection, and volume depletion events. (Sims et al., 2018). The overall safety profile of sotagliflozin is comparable to that of that of other SGLT2 inhibitors. (Avgerinos et al., 2022).

In addition to the differences in the reported clinical outcomes of sotagliflozin and dapagliflozin, it is worth that these medications also confer cost-effectiveness that may influence their benefits. Based on DAPA-HF, this study investigated the cost-effectiveness of dapagliflozin compared to a placebo among heart failure patients with diabetes. This finding demonstrated that dapagliflozin was projected to add 0.63 (95%uncertainty interval [UI], 0.25-1.15) quality adjusted life-years (QALYs), with an incremental lifetime ratio of $42,800 (95%UI, $37,100-$50,300), resulting in an incremental cost-effectiveness ratio of $68,300 per QALY gained (95%UI, $54,600-$117,600 per QALY gained). (Isaza et al., 2021). Conversely, the use of sotagliflozin incurred an incremental lifetime ratio of $19,374 and resulted in a net gain in QALYs of 0.425, with an estimated incremental cost-effectiveness ratio of $45,596 per QALY gained based on the SOLOIST-WHF trial. (Kim et al., 2023). So dapagliflozin was linked to a net increase of 0.205 QALYs compared to sotagliflozin, with a 33.2% lower cost per QALY gained. Hence, prescribing medication maybe based on the patient’s specific condition is clinically imperative.

Despite mounting evidence indicating that sotagliflozin is significantly superior to dapagliflozin in terms of both efficacy and affordability, its clinical use remains limited. This limitation partly stems from the uncertainty surrounding costs and partly from the lack of understanding of sotagliflozin. For instance, the literature we cited suggests that sotagliflozin demonstrates efficacy specifically in individuals with diabetes and worsening heart failure. This could provide healthcare professionals with the flexibility to tailor the medication to the patient’s condition during decision-making analyses.

### 4.1 Limitation

There are several limitations of this study. First, the experimental data in this analysis were obtained from the SOLOIST-WHF trial. The trial sponsor changed from Sanofi to Lexicon Pharmaceuticals in the middle of the trial, leading to alterations in some endpoints and related parameters, such as the median duration of follow-up. Moreover, the baseline values for enrollment of subjects in the two clinical trials were less homogeneous, potentially leading to some differences in the statistics of the data.

Secondly, due to the timing of the drug launch in the intervention group, we currently lack price data for these drugs. Therefore, this analysis employs sensitivity prediction analysis, using the drug price as a baseline in the control group to analyze the indicators of the intervention group.

Finally, and most importantly, this study does not replace cost-effectiveness analyses of medicines to achieve the QALYs. The CNT and NNT in this study are calculated from the patient’s median follow-up time and the odds of preventing a single event. However, using CNT and NNT for decision analyses of medicines has its limitations, as the number of treatments it requires varies with the length of follow-up. (Altman and Andersen, 1999). This explains the large difference in results between the control and intervention groups in this study. Moreover, NNT can only measure studies comparing different treatments for the same disease, (Pitt et al., 2014) i.e,., choosing the superior one of two comparable treatments. Nonetheless, NNT has been shown to be an objective, clinically relevant, descriptive, and easily interpretable measure of clinical data in several ways, particularly when applying trial results in a clinical setting, where annualized rates appear to be more effective than absolute risk reductions in assessing chronic disease. (Walter and Irwig, 2001; Greenstein and Nunn, 2004; Cazzola, 2006; Mayne et al., 2006).

## 5 Conclusion

In summary, dapagliflozin and sotagliflozin seem comparable in terms of safety in treating diabetes in individuals with heart failure. However, the preliminary results of this study suggest that sotagliflozin is more likely to significantly reduce the incidence of patients needed to prevent a single event and decrease medication expenses. Additionally, as a new class of SGLT-1/SGLT-2 dual-acting inhibitors, sotagliflozin markedly lowers glucose concentrations in the gastrointestinal tract. Therefore, this study supports including sotagliflozin as a therapeutic agent in relevant guidelines for treating heart failure.

## Data available statement

The original contributions presented in the study are included in the article/supplementary material, further inquiries can be directed to the corresponding author.
